# Differential induction of antioxidant and anti-inflammatory phytochemicals in agitated micro-shoot cultures of *Ajuga integrifolia* Buch. Ham. ex D.Don with biotic elicitors

**DOI:** 10.1186/s13568-021-01297-3

**Published:** 2021-10-18

**Authors:** Muhammad Asad Ullah, Faiza Zareen Gul, Taimoor Khan, Muhammad Naeem Bajwa, Samantha Drouet, Duangjai Tungmunnithum, Nathalie Giglioli-Guivarc’h, Chunzhao Liu, Christophe Hano, Bilal Haider Abbasi

**Affiliations:** 1 Department of Biotechnology, Quaid-i-Azam University, Islamabad, 45320 Pakistan; 2 School of Agriculture and Food Sciences, The University of Queensland, Gatton Campus, Brisbane, 4343 Australia; 3 Natural and Medical Sciences Research Centre, University of Nizwa, 616 Nizwa, Sultanate of Oman; 4 Laboratoire de Biologie des Ligneux et des Grandes Cultures (LBLGC), INRA USC1328, Université d’Orléans, 45067 Orléans Cedex 2, France; 5 COSM’ACTIFS, Bioactifs et Cosmétiques, CNRS GDR3711, 45067 Orléans Cedex 2, France; 6 Department of Pharmaceutical Botany, Faculty of Pharmacy, Mahidol University, Bangkok, 10400 Thailand; 7 EA2106 Biomolecules et Biotechnologies Vegetales, Universite Francois-Rabelais de Tours, Tours, France; 8 State Key Laboratory of Bio-Fibers and Eco-Textiles, Institute of Biochemical Engineering, College of Materials Science and Engineering, Qingdao University, Qingdao, 266071 People’s Republic of China

**Keywords:** Yeast extract, Pectin, Phytochemistry, Biological activities, In-vitro shoot culture, HPLC

## Abstract

*Ajuga integrifolia* Buch. Ham. ex D.Don, a member of *Lamiaceae* family is pharmaceutically an active perennial herb widely spread in China, Afghanistan and Pakistan Himalayan region. The application of biotic elicitors is a promising approach to cover limitations of in vitro cell technology and challenges faced by pharmaceuticals industry for bulk up production. The current study involved the induction of agitated micro-shoot cultures with the aim to investigate the growth-promoting as well as phytochemicals enhancement role of yeast extract (YE) and pectin (PE). The results showed that both elicitors induced a considerable physiological response. Biomass accumulation was observed maximum (DW: 18.3 g/L) against PE (10 mg/L) compared to YE and control. Eleven secondary phytocompounds were quantified using high-performance liquid chromatography. PE (50 mg/L) was found to be effective in elicitation of rosmarinic acid (680.20 µg/g), chlorogenic acid (294.12 µg/g), apigenin (579.61 µg/g) and quercetin (596.89 µg/g). However, maximum caffeic acid (359.52 µg/g) and luteolin (546.12 µg/g accumulation was noted in PE (1 mg/L) treatment. Harpagide, aucubin, harpagoside and 8-*O*-acetyl-harpagoside production was suppressed by both elicitors except for YE (100 mg/L). Catalpol accumulation in micro-shoot cultures was also downregulated except in response to YE (50 and 100 mg/L). Antioxidant activity and anti-inflammatory activity remained higher under PE (50 mg/L) and YE (100 mg/L) respectively. Therefore, results suggested that *Ajuga integrifolia* micro-shoot cultures treated with yeast extract and pectin might be an efficient bio-factory to produce commercially potent specific secondary metabolites.

## Key points

- In vitro agitated shoot cultures were established for the first time and exposed to biotic elicitors.
- Pectin exposure induced higher levels of rosmarinic acid, caffeic acid, apigenin and chlorogenic acid.
- Antioxidant and anti-inflammatory potential of *A. integrifolia* significantly increased with an increase in phytochemical contents.

## Introduction

**Fig. 1:**
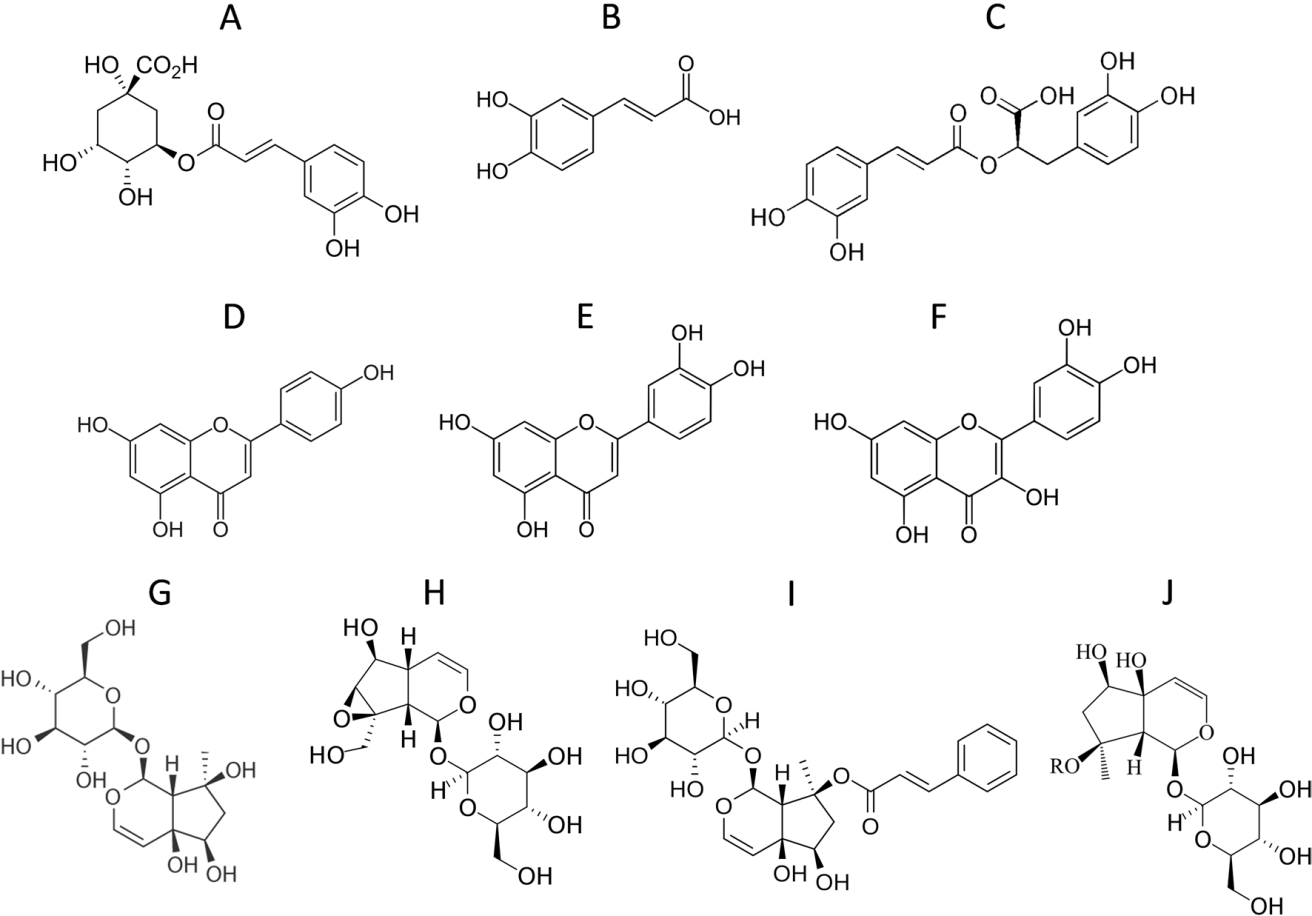
Chemical structures of the main phytochemicals from A. *integrifolia* analyzed by HPLC. **A** chlorogenic acid; **B** caffeic acid; **C** rosmarinic acid; **D** apigenin; **E** luteolin; **F** quercetin; **G** harpagide; **H** aucubin; **I** catalpol; **J** 8-*O*-acetyl-harpagoside (R = acetyl)

Medicinal plants opted for numerous uses in medicine, flavours, food, cosmetics and other industries (Zhao et al. 2005). Modern pharmaceutical industry is shifting towards traditionally used medicinal plants to mitigate the effects of synthetic drugs at the cellular level (Dar et al. 2017). *Ajuga* genus belongs to the family *Lamiaceae* and has almost 300 species of pharmaceutically active flowering plants (Israili and Lyoussi 2009). Among them *Ajuga integrifolia* Buch. Ham. ex D.Don is a perennial herb, grows 5–50 cm tall and is commonly known as Kauri booti in Pakistan due to vicious taste (Jan et al. 2014). It is widespread in Himalayan region of Asia including Pakistan, China and Afghanistan. *A. integrifolia* (synonym *Ajuga bracteosa*) contains various bioactive compounds including phytoecdysones, flavonol glycosides, ergosterol-5,8-endoperoxide, neo-clerodane diterpenoids and iridoid glycosides (Fig. 1). Because of these active compounds, *A. integrifolia* has intensively been used as an antimicrobial, anti-inflammatory, antiarthritic, cardiotonic, antimalarial, antitumor and antioxidant agent (Abbasi et al. 2020).

Isolation and analysis of plant secondary metabolites has gained huge attention in the last three decades due to their marvellous pharmacological activities (Kabera et al. 2014). It is declared as endangered species in the entire region cited in the International Union for Conservation of Nature Red List Database (Hussain et al. 2016; Saeed et al. 2017). To conserve endangered species, secondary metabolite production and develop diseases free plants, the tissue culture technique is a promising alternative irrespective of environmental limitations (Oseni et al. 2018). For instance, in vitro shoot organogenesis is a considerable method to conserve the plant by producing clones in a short time period (Verma et al. 2011). The adaptive response toward stress in plants via releasing secondary metabolites is an attractive approach for pharmaceutical industries where the production can be enhanced by employing elicitors (Giri and Zaheer 2016; Thakur and Sohal 2013). Elicitors are mainly classified into two types: “*biotic and abiotic*”. Biotic elicitors originate from biological sources either from microorganisms or plant cell walls, whereas abiotic elicitors are from non-biological origin starring physical, chemical and hormonal factors (Naik and Al-Khayri 2016).

Pectin is a natural component (polysaccharide) isolated from the cell walls of plant, particularly from citrus products having three structural motifs, i.e., rhamnogalacturonan I, rhamnogalacturonan II and homogalacturonan. Biocompatibility, biodegradability, non-toxicity and gelling property make it an efficient material to be used commercially in cosmetics, the food industry, the development of biomaterials and targeted drug delivery (Chen et al. 2015; Mishra et al. 2012). Pectin profound to be a competent elicitor from the biotic group in generating the defence response that boosts the secondary metabolites production (Zheng et al. 2020). Likely, yeast extract as a polysaccharide contains vitamin B-complex, glucans, chitin, ergosterols and glycopeptides that elicit the immune response in plants (Maqsood and Abdul 2017). Endogenously, yeast extract activates the metabolic pathways, mediates signal recognition and transduction that are necessary for biosynthesis of metabolites in plants (Namdeo 2007; Zhai et al. 2017). Our recent study showed the growth stimulatory effects of gibberellic acid and phytochemical stimulatory effects of salicylic acid in basil shoot culture of *A. integrioflia* (Abbasi et al. 2020). Therefore, to further assess how changing shoot culture methodology and elicitors affect growth and phytochemical induction in *A. integrioflia,* agitated micro-shoot culture was established in the current study. Different concentrations of pectin and yeast extract elicitors were applied in agitated cultures to analyze their effects on biological potential, i.e., anti-inflammatory and antioxidant, biomass accumulation, and phytochemicals production. Moreover, phytochemicals were quantified individually through high-performance liquid chromatography in agitated micro-shoots of *A. integrifolia*.

## Materials and methods

### Micro-shoot cultures of *Ajuga integrifolia*

Fresh leaves of *Ajuga integrifolia* were collected from wild-grown plants near the Department of Biotechnology, Quaid-i-Azam University Islamabad, to establish in vitro shoot culture. Surface sterilization of leaves was done for 40 s with 0.1% mercuric chloride, followed by 60 s washing with 70% ethanol and rinsed with autoclave distilled water thrice. After that, the leaves were sliced in size 0.5 cm^2^ approx. Inoculation of leaves on MS media (Murashige and Skoog 1962) were performed in laminar flow hood for establishing *A. integrifolia *in vitro shoot culture. To promote shooting, BAP (6-benzylaminopurine) of concentration 1.0 mg/L was added in MS media containing sucrose (3%) and agar (0.8%) as a solidifying agent. The pH of the medium was adjusted before autoclave between 5.6 to 5.8 by using basic (NaOH) and acidic (HCl) solution. Media was poured in conical flasks (100 ml) and autoclaved for 20 min at 121 °C, 15 psi. Inoculated flasks for maximum biomass production were placed in a growth chamber under dark conditions at 25 °C ± 2 °C.

### Treatment of yeast extract (YE) and pectin (PE) in agitated micro-shoot culture

Agitated shoot culture was established in 100 ml Erlenmeyer flasks from *A. integrifolia* shoot cultures grown in dark according to Abbasi et al. (2020). In short, MS media was prepared under aseptic conditions without any solidifying agent containing different concentrations of biotic elicitors i.e., yeast extract (1.0, 10, 50, 100, 200 mg/L) and pectin (1.0, 10, 50, 100, 250, 500 mg/L). The stock solution of yeast extract was prepared according to Zhao et al. (2014) and subsequently precipitates were used for the experiment in concentration-dependent manner. Gyratory shakers were used at constant agitation (120 rpm) in growth room (25 ± 2 °C) for agitated micro-shoot cultures establishment. Shoots without any treatment were considered as control.

### Sample extraction

Cultures were harvested after 3 weeks of treatment and placed on Whatman filter paper for removal of extra water contents. Fresh weight of micro-shoots was measured, and cultures were placed at 45˚C in a fan forced oven for 1–2 days until constant change in weight was achieved to calculate the dry weight. Dried cultures were ground into a fine powder and, according to Ullah et al. (2019a), were subjected to extraction. Briefly, sample (0.1 g) was homogenized in methanol (0.5 ml), vortexed and sonicated twice for 5 min and 30 min, respectively. Furthermore, samples were centrifuged at12,000 rpm for 10 min. The supernatant was separated in a sterile Eppendorf tube and placed in a refrigerator at 4 °C for in vitro biological activities and phytochemical analysis later.

### Phytochemical estimation of *Ajuga integrifolia*

#### Total phenolic and flavonoids

According to the protocol of Singleton and Rossi (1965), total phenolic contents (TPC) of micro-shoots were determined. In short, sample extract (20 µl) was added in 96 well plates containing a mixture of 90 µl of Folin–Ciocalteu (FC) reagent and 90 µl Na_2_CO_3_. Following 5 min incubation at 25 °C, the reaction mixture was subjected to Synergy II microplate reader and absorbance reading were noted at 725 nm wavelength. Positive control in qualitative phenolic estimation was gallic acid, while DMSO was the negative control. Results were expressed as gallic acid equivalents (GAE)/g of dry weight. Total phenolic production (TPP) was calculated as,

$${\text{TPP }}\left( {{\text{mg}}/{\text{L}}} \right) \, = {\text{ dry weight }}\left( {{\text{g}}/{\text{L}}} \right) \, \times {\text{ total phenolic contents }}\left( {{\text{mg}}/{\text{g}}} \right)$$

The protocol established by Abbasi et al. (2011) was used to calculate the total flavonoid contents (TFC). Briefly, sample (20 µl) was taken in 96 well microplates containing distilled water (160 µl), potassium acetate (10 µl), and aluminium chloride (10 µl) in each well. Following 30 min incubation at 25 °C, the reaction mixture was subjected to a microplate reader and absorbance reading was noted at 630 nm wavelength. Quercetin was used as a standard in TFC estimation and the results were calculated as quercetin equivalents (QE)/g of dry weight. Total flavonoid production (TFP) was calculated by using the following formula,

$${\text{TFP }}\left( {{\text{mg}}/{\text{L}}} \right) \, = {\text{ dry weight }}\left( {{\text{g}}/{\text{L}}} \right) \, \times {\text{ total flavonoid contents }}\left( {{\text{mg}}/{\text{g}}} \right)$$

#### Quantitative estimation of polyphenols via HPLC

High-performance liquid chromatography was performed for the quantification of phytochemicals accumulated in micro-shoot cultures of *A. integrifolia*. Galaxie version 1.9.3.2 software with Varian HPLC system (Varian Prostar 230 pump, Varian Prostar 410 autosampler and Varian Prostar 335 Photodiode Array Detector) was used to separate the components. Reading was measured at 320 nm. Two HPLC grade solvents i.e., HCOOH/ H_2_O, pH = 2.1 (A) and CH_3_OH (B) were used in the mobile phase with a flow rate 1 ml/min. Composition of mobile phase varied according to nonlinear gradient i.e., 8% B (0 min), 12% B (11 min), 30% B (17 min), 33% B (28 min), 100% B (30– 35 min), 8% B (36 min). Ten min of equilibration time after each run was applied. Phytochemicals were quantified based on retention times and UV spectra compared with authentic standards.

### In vitro antioxidant activities

*DPPH free radical scavenging activity* To check the *A. integrifolia* micro-shoots scavenging activity, sample (20 µl) was mixed with DPPH solution (180 µl) and incubated in the dark for 1 h, according to Ullah et al. (2019b). The absorbance of the reaction mixture was measured at 517 nm by using a microplate reader. Negative control in DPPH activity was DMSO (20 µl) while ascorbic acid (20 µl) is used as a positive control. The following formula was used to calculate DPPH scavenging activity:

$$\% {\text{ scavenging activity of free radicals }} = \, \left( {{1 } - {\text{ absorbance value with samples}}/{\text{absorbance value without sample}}} \right) \, \times { 1}00$$

*Ferric reducing antioxidant power (FRAP) assay* FRAP method described by Benzie and Strain (1996) was used to measure the antioxidant capacity of *A. integrifolia* micro-shoot cultures. In brief, FRAP solution (190 µL), composed of 10 Mm TPTZ, 300 mM acetate buffer and 20 mM ferric chloride hexahydrate in the respective ratio of 1:10:1 (v:v:v), was mixed with sample extract (10 µL) and incubated at 25 ± 2 °C for 15 min. The absorbance of the testing sample was measured at 630 nm wavelength by a microplate reader. Results were expressed as Trolox C equivalent antioxidant capacity (µM TEAC).

*ABTS antioxidant assay* Tagliazucchi et al. (2010) method was employed to measure the antioxidant potential of *A. integrifolia* agitated micro-shoot cultures. ABTS salt (2,2-azinobis (3-ethylbenzthiazoline-6-sulphonic acid, 7 mM) and potassium per sulphate (2.45 mM) was mixed to prepare ABTS solution and incubated in the dark for 16 h. Sample and ABTS solution was mixed at room temperature and incubated for 15 min. After that, absorbance was taken at 734 nm by a microplate reader. Antioxidant ABTS assay results were denoted as TEAC (Trolox C equivalent antioxidant capacity).

### In vitro anti-inflammatory activities

#### COX-1 and COX-2 inhibitory activity

Inhibitory activity of shoot extracts against cyclooxygenase enzymes (COX-1 (Ovine) and COX-2 (human)) was determined by using assay kit (701050, Cayman Chem. Co, Interchim, Montluçon, France). 10 μM of ibuprofen were used as positive control and 1.1 mM of arachidonic acid as substrate. Oxidation of *N,N,N′,N′-*tetramethyl-*p*-phenylenediamine were measured for 5 min using Synergy II reader at 590 nm.

#### 15-LOX inhibitory activity

Inhibitory activity of *Ajuga integrifolia* micro-shoot extracts against 15-LOX was analyzed by using an assay kit (760700, Cayman Chem. Co, Interchim, Montluçon, France). Ten μM of arachidonic acid was used as positive control and 100 μM of nordihydroguariaretic acid act as a substrate. Hydroperoxides concentration in the lipoxygenation reaction was measured. Absorbance was measured by using Synergy reader at 940 nm. Inhibitor and enzyme were incubated for 5 min and absorbance was recorded. Afterwards, absorbance was measured after addition and incubation of substrate for 15 min followed by addition of chromogen and incubation for 5 min.

#### Secretory phospholipase A2 (sPLA2) inhibitory activity

sPLA2 inhibitory activity was measured by using assay kit and manufacturer guidelines (10004883, Cayman Chem. Co, Interchim, Montluçon, France). 100 μM of thiotheramide-PC as positive control, while Diheptanoyl thio-PC of concentration 1.44 mM were used as substrate in this reaction. Absorbance was measured using a microplate reader at 420 nm which measured free thiols releases by diheptanoyl thio-PC ester cleavage. Results were calculated as

$$\% {\text{ Inhibition }} = \, \left[ {\left( {\text{IA}}-{\text{Inhibitor}} \right)/{\text{IA}}} \right] \, \times { 1}00$$

Here, ‘IA’ refers to enzymatic activity without inhibitor whereas ‘Inhibitor’ refers to enzymatic activity with inhibitor.

### Statistical analysis

All of the experiments discussed in this paper were conducted in triplicates and repeated twice. Microsoft Excel software was used to calculate mean ± standard deviation. Graphs were designed by using origin software. One-way analysis of variance (ANOVA) was used, and means were separated at significant difference p < 0.05.

## Results

### Biomass production of *A. integrifolia* agitated micro-shoots

**Fig. 2:**
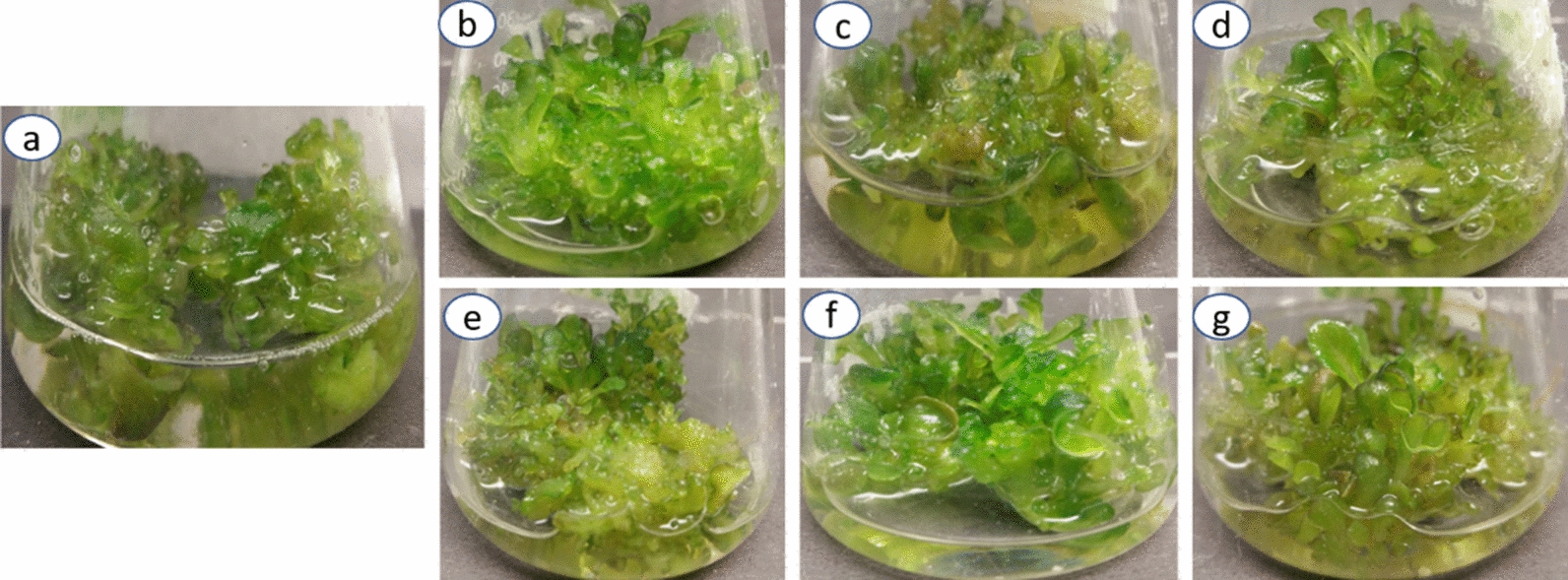
Morphology of *Ajuga integrifolia* agitated micro-shoot cultures, **a** control, **b** YE 1.0 mg/L, **c** YE 10 mg/L, **d** YE 200 mg/L, **e** PE 1.0 m/L, **f** PE 10 mg/L, **g** PE 200 mg/L

**Fig. 3:**
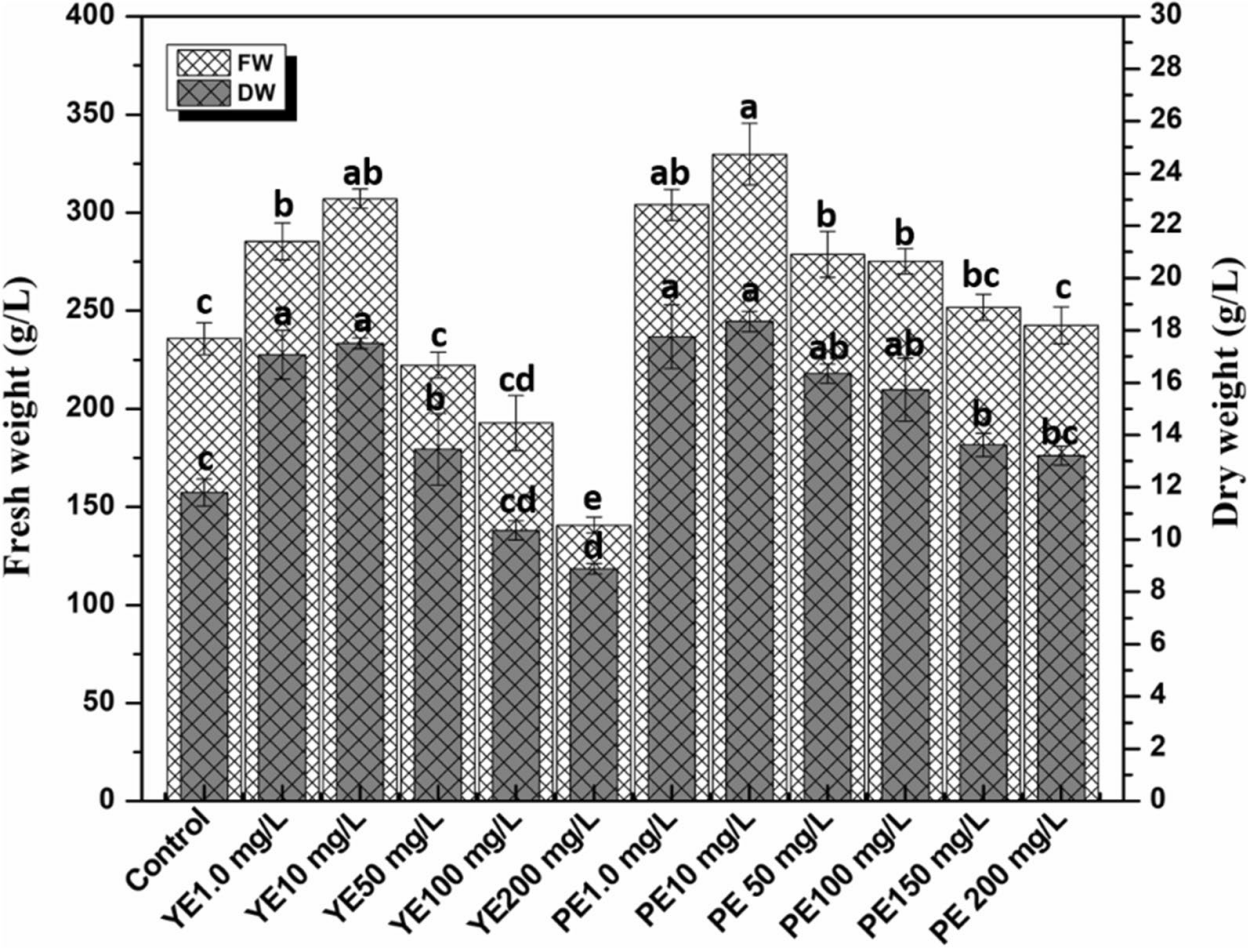
Biomass accumulation in *Ajuga integrifolia* agitated micro-shoot cultures in response to yeast extract (YE) and pectin (PE). Values represent means ± standard errors from triplicates; different letters indicate significant differences at p < 0.05

In the current study, biomass accumulation was recorded in vitro agitated micro-shoot cultures elicited with various concentrations of YE and PE for 3 weeks, as shown in Figs. 2 and 3. Overall, PE treated micro-shoot cultures showed active biomass accumulation than YE and control. Maximum fresh weight was recorded in PE (10 mg/L, FW: 329.8 g/L) as compared to YE and control cultures. Agitated micro-shoot cultures treated with YE showed increased biomass at concentration 1.0–50 mg/L comparative to control (FW 235.7 g/L). A similar trend was found in dry weight in both elicitors’ treatments, and PE (10 mg/L) treated cultures had higher dry weight (18.3 g/L) in the overall designed experiment.

### Phytochemical production in *A. integrifolia* micro-shoot cultures

**Fig. 4:**
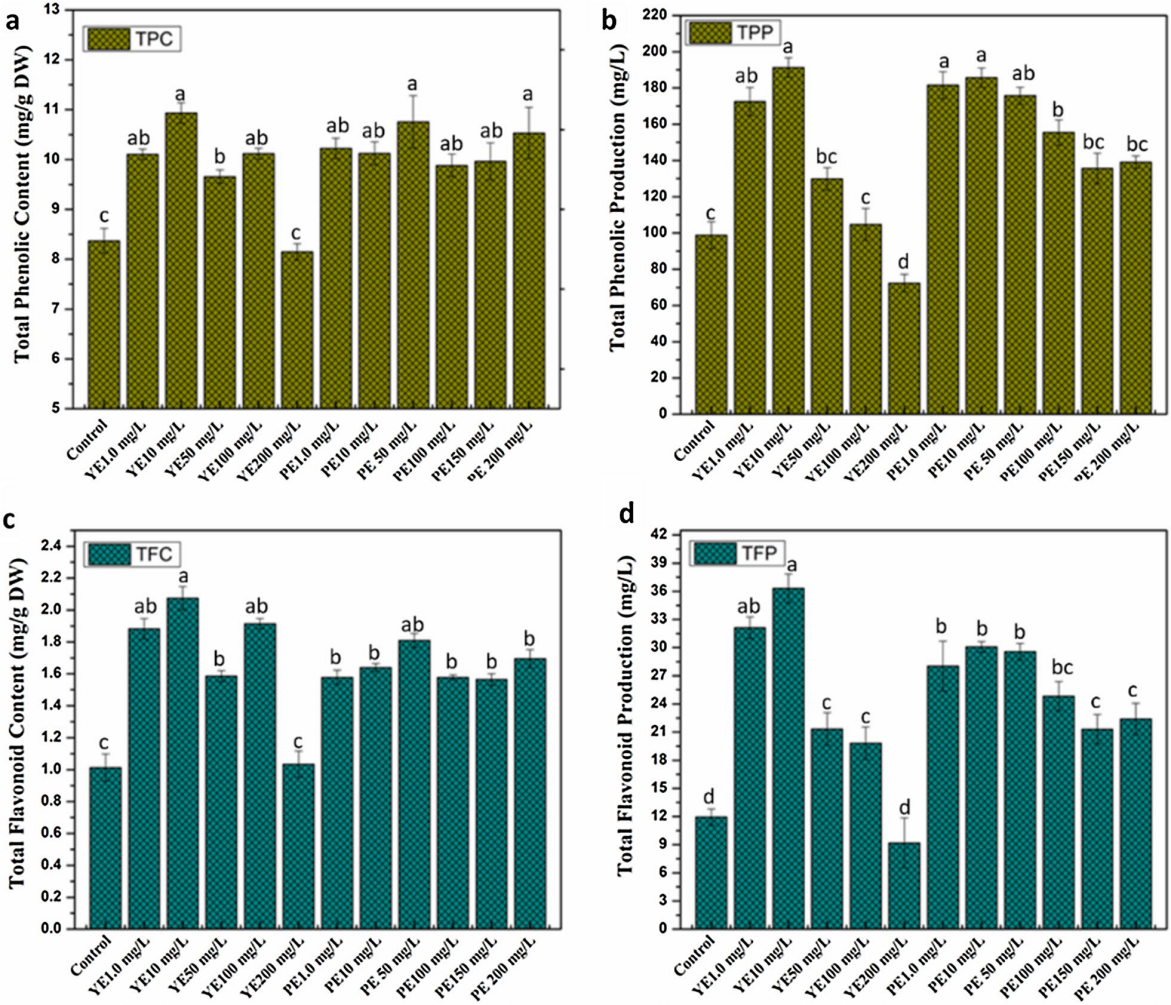
Impact of yeast extract (YE) and pectin (PE) on: **a** Total phenolic contents (TPC), **b** Total phenolic production (TPP), **c** Total flavonoid contents (TFC), **d** Total flavonoid production (TFP). Values represent means ± standard errors from triplicates; different letters indicate significant differences at p < 0.05

Phytochemicals are defensive agents produced by plants against various biotic or abiotic stress conditions. In the current study, total phenolic and flavonoid contents of micro-shoots culture were investigated against different concentrations of YE and PE. Both elicitors have shown significant results on phytochemical contents and their production relative to control (Fig. 4). Maximum phenolic contents (TPC 10.9 mg/g) were noted in YE (10 mg/L) treatment followed by TPC 10.7 mg/g at PE (50 mg/L) comparative to control (8.36 mg/g) (Fig. 4a). Total phenolic production results were calculated based on dry weight and TPC (Fig. 4b). Increased in TPP was observed in cultures supplemented with YE at all concentrations except 200 mg/L (98.7 mg/L) comparative to control (98.7 mg/L). Overall maximum phenolic production (TPP 191.2 mg/L) was noted in YE (10 mg/L) treated cultures which was correlated with total phenolic contents. Following the results of TPC, flavonoid contents were also increased against YE and PE treatments in micro-shoot cultures (Fig. 4c). Optimum flavonoid contents (TFC 2.07 mg/g) were observed in YE (10 mg/L) followed by 1.80 mg/g for PE (50 mg/L). The trend observed in TFP was similar to TPC, TPP and TFC as highest TFP (36.3 mg/L) was observed in response to YE (10 mg/L) treated cultures in comparison with PE and control (11.9 mg/L) (Fig. 4d).

### Phytochemical quantification via HPLC

High-performance liquid chromatography was employed to quantify individual phytochemical compounds accumulated in agitated micro-shoot cultures of *A. integrifolia*. Total 11 phytochemical compounds were quantified against YE and PE stress (Table 1). Optimum levels of rosmarinic acid (680.2 µg/g DW), chlorogenic acid (294.1 µg/g DW), apigenin (579.6 µg/g DW) and quercetin (596.8 µg/g DW) were noted in cultures treated with PE (50 mg/L) correlated with the findings of phytochemical and antioxidant activities. Among all YE treatments, chlorogenic acid, caffeic acid, rosmarinic acid and quercetin accumulation was maximum in cultures supplemented with YE (10 mg/L) interrelated with antioxidant activity and phytochemical production. However, PE (1 mg/L) treated cultures have shown maximum accumulation of caffeic acid (359.5 µg/g DW) and luteolin (546.1 µg/g DW). Micro-shoots supplemented with both elicitors downregulated the production of harpagide, aucubin, harpagoside and 8-*O*-acetyl-harpagoside except in YE (100 mg/L) treatment when compared to control. Catalpol accumulation was also downregulated in all YE and PE treatments except in 50 and 100 mg/L (YE) comparative to control.

**Table 1:** HPLC analysis of phytochemical compounds in agitated micro-shoot cultures of *Ajuga integrifolia*

Treatment	Conc	Phytochemicals quantification via HPLC (in µg/g DW)										
		Chlorogenic Acid	Caffeic Acid	Rosmarinic Acid	Apigenin	Luteolin	Quercetin	Harpagide	Aucubin	Harpagoside	Catalpol	8-*O*-acetyl-harpagoside
Control	–	165.11 ± 2.48	200.17 ± 1.16	386.41 ± 4.67	483.47 ± 3.55	483.47 ± 2.31	501.02 ± 4.66	303.58 ± 5.33	166.03 ± 2.96	147.69 ± 4.46	101.85 ± 2.55	211.88 ± 3.21
Yeast extract (mg/L)	1	187.70 ± 5.34*	232.82 ± 4.25***	428.33 ± 3.46***	508.52 ± 2.34*	474.10 ± 6.26	508.52 ± 2.71	292.13 ± 3.17	155.65 ± 4.34	137.45 ± 2.70*	101.06 ± 2.41	192.04 ± 2.34*
	10	214.59 ± 2.16***	256.47 ± 2.84***	494.51 ± 3.54***	504.01 ± 4.61*	486.35 ± 2.58	539.32 ± 4.93*	253.01 ± 1.92**	141.00 ± 2.62*	122.34 ± 3.92**	94.34 ± 3.07	178.34 ± 4.58**
	50	182.31 ± 4.79**	225.53 ± 1.76***	413.55 ± 4.24***	459.56 ± 6.52*	494.18 ± 4.96	494.18 ± 3.09	293.83 ± 4.84*	165.71 ± 3.75	147.41 ± 4.23	110.80 ± 6.48	202.32 ± 4.67
	100	180.50 ± 6.73**	210.82 ± 1.95**	410.03 ± 5.16***	477.80 ± 3.86	477.80 ± 3.71	495.15 ± 1.82	335.01 ± 2.76**	186.56 ± 2.98**	168.00 ± 1.45**	121.61 ± 2.64**	242.23 ± 2.49**
	200	213.40 ± 3.46***	252.45 ± 4.84***	488.93 ± 2.58***	513.50 ± 5.94*	496.12 ± 1.02	513.50 ± 2.46*	249.05 ± 6.52***	138.80 ± 2.41**	120.42 ± 5.63**	92.86 ± 1.77	175.55 ± 2.85**
Pectin (mg/L)	1	281.72 ± 4.75 ***	359.52 ± 3.73 ***	668.56 ± 1.52 ***	563.43 ± 2.85 ***	546.12 ± 1.07 **	580.74 ± 5.57 ***	174.86 ± 3.06 ***	101.65 ± 4.56 ***	92.50 ± 2.50 ***	65.04 ± 3.28 **	129.10 ± 1.72 ***
	10	254.48 ± 3.52***	312.04 ± 2.91***	593.47 ± 2.34***	521.69 ± 2.52**	504.61 ± 2.81**	538.77 ± 2.84**	199.59 ± 1.46***	118.34 ± 1.04***	100.28 ± 6.17**	73.19 ± 4.64*	136.39 ± 2.93***
	50	294.12 ± 0.24***	343.72 ± 6.84***	680.20 ± 4.19***	579.61 ± 4.46***	545.06 ± 1.46**	596.89 ± 4.92***	174.52 ± 2.40***	101.45 ± 2.84***	83.18 ± 3.84***	64.91 ± 5.97**	128.85 ± 5.46**
	100	192.93 ± 1.13***	228.82 ± 4.25**	425.21 ± 3.73***	499.81 ± 6.01	449.06 ± 4.07**	499.81 ± 6.03	269.23 ± 2.08***	152.98 ± 3.53*	135.10 ± 2.14*	99.32 ± 2.23	188.75 ± 2.57*
	250	202.76 ± 3.36***	255.45 ± 3.59***	455.23 ± 5.84***	537.19 ± 7.16**	484.43 ± 2.72	519.60 ± 3.14*	270.60 ± 4.79***	149.74 ± 3.26*	131.15 ± 1.57*	93.96 ± 4.52*	186.93 ± 4.14*
	500	240.56 ± 2.12***	297.75 ± 2.64***	557.32 ± 2.78***	520.63 ± 3.89**	520.63 ± 3.18**	538.25 ± 1.05**	224.56 ± 1.43***	122.09 ± 4.74***	112.78 ± 2.92**	84.83 ± 2.44**	150.04 ± 3.64**

Values represent means ± standard errors from triplicates; *p < 0.05, **p < 0.01, ***p < 0.001 compared to control

### Antioxidant activity of *A. integrifolia* micro-shoot cultures

Plants produce excessive reactive oxygen species (ROS) in stress conditions that induce DNA damages, disrupt normal metabolism and affect growth (Huang et al. 2019). To scavenge these cell-damaging radicals, cellular machinery produce a wide range of enzymatic and non-enzymatic antioxidants to reduce ROS and improve stress tolerance (Das and Roychoudhury 2014). Antioxidant activity comprising a single assay is not fruitful as plant extract contain a variety of secondary metabolites with their unique pathway to express antioxidant potential (Prior et al. 2005). For analyzing antioxidant activity of *Ajuga integrifolia* micro-shoot cultures against PE and YE stress, in vitro antioxidant assays i.e., DPPH (2,2-diphényl-1-picrylhydrazyle), ABTS (2,2-azino-bis-3-ethylbenzothiazoline-6-sulphonic acid) and FRAP (ferric reducing antioxidant power) were performed in the current study. DPPH method follows the ET (electron transfer) and HAT (hydrogen atom transfer) mechanism, whereas ABTS along with FRAP based on HAT and ET mechanism respectively (Abbasi et al. 2020). Results exhibited that both elicitors induced potent antioxidant response comparative to control (Table 2). Maximum DPPH activity (89.8%) was shown by YE (10 mg/L), followed by PE (50 mg/L; 89.6%) comparative to control (81.2%). YE and PE induced significant DPPH response except in pectin 100 and 250 mg/L treatment. PE at high concentration (500 mg/L) showed enhanced DPPH activity (88.2%) as compared to control. Wherein, micro-shoots treated with PE (50 mg/L) showed the highest ABTS activity (487.2 μM), followed by YE (10 mg/L; 340.1 μM) compared to control. PE had a higher ABTS activity response in the medium compared to YE treatments. Similarly, FRAP activity was found higher (790.3 μM) in PE (50 mg/L) treatment and 552 μM in YE (10 mg/L) mediated cultures than in control.

**Table 2:** Antioxidant activity of *Ajuga integrifolia* micro-shoot cultures. DPPH (2,2-diphényl-1-picrylhydrazyle), ABTS (2,2-azino-bis-3-ethylbenzothiazoline-6-sulphonic acid) and FRAP (ferric reducing antioxidant power)

Treatment	Concentration	Antioxidant activities		
		DPPH (%)	ABTS (TEAC μM)	FRAP (TEAC μM)
Control	–	81.2 ± 3.28^c^	245.23 ± 8.79 ^f^	397.77 ± 13.34^g^
Yeast extract (mg/L)	1	86.4 ± 2.14^b^	297.68 ± 4.67 ^de^	482.78 ± 19.46^e^
	10	89.8 ± 1.61^a^	340.41 ± 11.67 ^c^	552.21 ± 15.46^d^
	50	88.6 ± 2.46^a^	286.32 ± 23.48 ^e^	463.96 ± 11.67^ef^
	100	88.4 ± 1.42^a^	282.85 ± 13.45 ^e^	458.77 ± 12.46^f^
	200	85.4 ± 3.47^b^	337.64 ± 9.49 ^c^	547.67 ± 16.19^d^
Pectin (mg/L)	1	88.4 ± 0.97^a^	473.25 ± 8.49 ^a^	767.64 ± 11.34^a^
	10	85.3 ± 2.15^b^	418.47 ± 14.67 ^b^	678.74 ± 17.15^b^
	50	89.6 ± 1.35^a^	487.27 ± 12.78 ^a^	790.36 ± 13.64^a^
	100	82.2 ± 4.31^bc^	301.61 ± 21.25 ^d^	489.27 ± 15.17^e^
	250	82.5 ± 3.94^bc^	316.45 ± 16.31 ^d^	513.27 ± 9.67^de^
	500	88.2 ± 2.44^a^	384.12 ± 11.66 ^bc^	622.94 ± 14.97^c^

Values represent means ± standard errors from triplicates; different letters indicate significant differences at p < 0.05

### Anti-inflammatory activity of *A. integrifolia* micro-shoot cultures

Phytochemicals inhibit the action of inflammatory enzymes and work as a natural substitute in the pharmaceutical industry (Tungmunnithum et al. 2018). In the present study, micro-shoot extracts of *Ajuga integrifolia* were analyzed for anti-inflammatory activity by in vitro enzymatic % inhibition assays produced in inflammation (Table 3). Micro-shoots treated with YE (100 mg/L) showed optimum inhibition against COX1 (52.2%), COX2 (38.5%), 15-LOX (59.3%) and sPLA2 (64.5%) as compared to control and PE (Table 3). PE treated micro-shoots significantly decreased anti-inflammatory potential comparative to control. Among different treatments, PE (100 mg/L) showed higher % inhibition against inflammatory enzymes. However, results indicated that YE supplemented media in *A. integrifolia* micro-shoot cultures showed the stimulatory effect on anti-inflammatory potential than PE.

**Table 3:** Anti-inflammatory activity of *Ajuga integrifolia* agitated micro-shoots (Cyclooxygenases (COX1, COX2); Secretory phospholipase A2 (sPLA2); Lipoxygenase (15-LOX))

Treatment	Concentration	Anti-inflammatory activities (% Inhibition)			
		COX1	COX2	15-LOX	sPLA2
Control	–	45.26 ± 2.31^b^	33.40 ± 1.02^ab^	51.46 ± 3.19^b^	55.95 ± 2.09^b^
Yeast extract (mg/L)	1	43.01 ± 1.05^b^	31.74 ± 3.76^b^	48.90 ± 2.42^b^	53.17 ± 1.87^b^
	10	37.91 ± 4.01^bc^	27.98 ± 1.43^bc^	43.10 ± 1.13^bc^	46.87 ± 2.61^c^
	50	44.76 ± 2.24^b^	33.03 ± 1.27^ab^	50.88 ± 0.88^b^	55.32 ± 3.13^b^
	100	52.20 ± 1.31^a^	38.52 ± 0.79^a^	59.35 ± 1.13^a^	64.53 ± 2.25^a^
	200	37.60 ± 3.56^bc^	27.75 ± 3.14^bc^	42.75 ± 3.02^bc^	46.48 ± 1.13^c^
Pectin (mg/L)	1	27.05 ± 0.72^cd^	19.96 ± 2.61	30.75 ± 2.65^d^	33.44 ± 2.25^de^
	10	30.59 ± 1.38^c^	22.57 ± 2.44^c^	34.78 ± 3.63^cd^	37.82 ± 3.41^d^
	50	26.27 ± 0.94^cd^	19.39 ± 1.83^d^	29.87 ± 1.22^d^	32.48 ± 2.72^de^
	100	42.44 ± 1.61^b^	31.32 ± 1.68^b^	48.25 ± 1.63^b^	52.46 ± 1.37^bc^
	250	40.46 ± 1.44^bc^	29.85 ± 0.95^b^	45.99 ± 2.24^bc^	50.01 ± 2.01^bc^
	500	33.33 ± 1.75^c^	24.60 ± 1.28^bc^	37.90 ± 1.96^c^	41.21 ± 0.99^cd^

Values represent means ± standard errors from triplicates; different letters indicate significant differences at p < 0.05

## Discussion

The impact of elicitor is highly dependent on plant species, elicitor and its concentration (Cai et al. 2012). In the current study, pectin stimulated biomass accumulation maybe because PE provides tensile strength to maintain the turgor pressure within the cell and increase the cell growth (Wolf and Greiner 2012). Stimulatory effects of PE agreed with the study of Wiktorowska et al. (2010) whose results on *Calendula officinalis* cell cultures indicated the increased cell growth and accumulation of oleanolic acid at low pectin concentration. However, yeast extract affects biomass accumulation by stimulating primary metabolism as amino acids and vitamins are the major constituents in YE (George et al. 2008). Previously, YE positively influenced biomass and lignans accumulation in bioreactor grown micro-shoot **c**ultures of *Schisandra chinensis* (Szopa et al. 2018). Biomass accumulation was enhanced in response to YE in cultures of *Linum usitatissimum* (Nadeem et al. 2018), *Tropaeolum majus* (Wielanek and Urbanek 2006), and *Scutellaria lateriflora* (Wilczańska-Barska et al. 2012). On the other hand, phenolics have no direct relation with plant growth and development, however, they act as a defensive compound in either biotic or any abiotic stress conditions (Dias et al. 2016). Both elicitors i.e., PE and YE, act as signalling molecules in induction of defensive response and increased the metabolite content previously in *S. rebaudiana *in vitro shoot cultures (Bayraktar et al. 2016). Pectin as an elicitor was found to be a promising candidate for improving phenylpropanoids in *Hypericum perforatum* cultures (Shakya et al. 2019) by increasing enzymatic activity of phenyl ammonia lyase at the 14th day of post elicitation. Additionally, suppressed Chalcone-flavanone isomerase activity was also noted, which inhibits flavonoid production (Gadzovska Simic et al. 2014). Previous reports indicated that chalcone isomerase gene expression directly correlates with flavonoid accumulation (Zhang et al. 2009). Increased flavonoid production may be due to the overexpression of flavonoid biosynthetic genes. Natural polysaccharide pectin present in plant cell walls improved the biomass accumulation and secondary metabolite stevioside content in in vitro micro-propagated *Stevia rebaudiana* cultures (Bayraktar et al. 2016). Our results corroborate the study of Al-Khayri and Naik (2020) on *Phoenix dactylifera* where PE (50 mg/L) enhanced the flavonoid contents in cell cultures. Similarly, Cai et al. (2012) found enhanced production of phenolic acids and anthocyanin upon exposure to pectin in *Vitis vinifera* cell cultures (Cai et al. 2012). YE has been an efficient elicitor in the production of secondary metabolites by increasing the proteins involved in secondary metabolism in in vitro derived cultures of different species. It contains *N*-acetyl glucosamine oligomers, chitin, ergosterol and glycopeptides which elicit the potent protective response against stress in plants (Ferrari 2010; Narayani and Srivastava 2017). YE upregulate the activity of phenylalanine-ammonia lyase, the first enzyme in the phenylpropanoid pathway in the biosynthesis of phenolics (Sarkate et al. 2017). In *Agastache rugosa* cell suspension culture, YE increased the transcript levels of RAS and HPPR genes that are involved in the phenylpropanoid biosynthetic pathway (Park et al. 2016). In parallel to our results, YE at low concentration efficiently enhanced the flavonoid production in hairy root cultures of *Pueraria candollei* and *Psoralea corylifolia* (Shinde et al. 2009; Udomsuk et al. 2011). In contrast, total phenolic and flavonoid contents were increased in cell suspension cultures of *Ocimum basilicum* supplemented with YE at 200 mg/L (Açıkgöz 2020) which again points that elicitor activity is highly dependent on plant species, elicitor and its concentration. Al Khateeb et al. (2017) reported enhanced phenolic production along with new phenolic compounds that were not initially present in control plants in micro-shoots of *Rumex cyprius* from 50 to 200 mg/L YE concentration in the medium. Elicitation with YE has successfully increased caffeic acid in *Echinacea puprea* callus cultures (Rady et al. 2018), asiaticoside in *Centella asiatica* and plumbagin in *Drosera burmanii* whole plant cultures (Kim et al. 2004; Putalun et al. 2010) and mitragynine in shoot cultures of *Mitragyna speciose* (Wungsintaweekul et al. 2012). Per our results, YE (50 and 100 mg/L) downregulated the apignenin production, while in the contrary, decreased the caffeic acid and apignenin accumulation in the presence of PE and YE in *Phoenix dactylifera* cell suspension cultures (Al-Khayri and Naik 2020). Phytochemicals quantified in *A. integrifolia* micro-shoot cultures have pharmaceutical importance, especially as antioxidant agents, disrupting cancer cellular machinery, suppression of oncogenes and apoptosis of cell (Mohammad Nabavi et al. 2015; White et al. 1989). In our study, increased antioxidant activity in response to elicitors is likely due to antioxidant phytochemicals and significant interactions between them in the presence of pectin in micro-shoot cultures of *A. integrifolia*. Pectin has an impact on binary combinations of phenolic compounds by using isobolograph analysis and combination index for DPPH and FRAP antioxidant analysis (Mercado-Mercado et al. 2020).

Inflammation is the immune response triggered by noxious stimuli within the cell and prostaglandins are key players in developing inflammation. Cyclooxygenase enzymes (COX-1 and COX-2) mainly converts arachidonic acid into prostaglandins (PGs) in the inflammatory pathway of producing physiological or disease response (Ricciotti and FitzGerald 2011). Lipoxygenases (12/15-LOX) generate various lipid bioactive mediators, including PGs, by taking arachidonic acid as substrate in the inflammatory process (Ackermann et al. 2017; Haeggström and Funk 2011). Secretory phospholipase A2 (sPLA2) is a lipolytic enzyme generally upregulated that enhanced arachidonic acid (AA) and prostaglandins (PGs) during inflammation (Hamaguchi et al. 2003). Non-steroidal anti-inflammatory drugs (NSAIDS) inhibits the synthesis of prostaglandins by blocking the action of these enzymes (Dwivedi et al. 2015). However, NSAIDS have adverse effects on cellular physiological functions (Bacchi et al. 2012). Our findings suggest an inter-connection of phytochemicals with anti-inflammatory potential of micro-shoot cultures treated with 100 mg/L of PE and YE (Tables 1, 3). In the current study, YE (100 mg/L) treatment was ideal in agitated micro-shoot cultures of *A. integrifolia* to provoke the anti-inflammatory response, possibly through increase phytochemical accumulation (Table 1). Ali et al. (2019) found that chloroform: methanol extract of *Zingiber officinale* callus cultures treated with YE (100 mg/L) significantly inhibited pro-inflammatory cytokines (Interleukin 1 (IL-1), Interlukin 6 (IL-6) and tumor necrosis factor α (TNF-α)) that modulates the activity of *β*-d-glucans present in yeast extract. Several *β*-d-glucans have previously been proved to be a potent polysaccharide to induce the anti-inflammatory response in both in vitro and in vivo studies (Du et al. 2015). On the other hand, immune-modulatory activity of PE depends on the structural characteristics of linear and branched chains of polysaccharides (Beukema et al. 2020). A high quantity of galacturonic acid in the pectin backbone inhibits its immune protective or anti-inflammatory function (Popov and Ovodov 2013). In this study, the reduced inflammatory activity of *A. integrifolia* micro-shoots could be due to the hindrance of pectin with cell structure and conformation vice versa. Although Gautam et al. (2011) reported that *Ajuga integrifolia* whole plant extract inhibited COX isoforms (COX-1 and COX-2) that supports the anti-inflammatory activity results (Table 3) of *A. integrifolia* micro-shoot cultures alone. Previously, multiple shoot cultures of *A. integrifolia* have shown anti-inflammatory potential against salicylic acid and gibberellic acid stress (Abbasi et al. 2020).

In summary, yeast extract and pectin exhibited stimulatory effects on cellular metabolism. Results revealed that pectin showed a preeminent response on biomass accumulation compared to control and yeast extract treatment. Total phenolic and flavonoid contents along with their production were maximum in cultures treated with yeast extract. Antioxidant activities were significantly higher in pectin treated micro-shoot cultures, whereas yeast extract treatment stimulated anti-inflammatory response. HPLC quantification of compounds was also found correlative with antioxidants and anti-inflammatory activities. Hence the findings of the current study strongly emphasize on the antioxidant and biological potential of *Ajuga integrifolia* micro-shoot cultures elicited with YE and PE. This study is significantly a contribution to the field that decodes possible directions towards sustainable production of medicinally relevant metabolites on a commercial level.

## Data Availability

The data will be made available upon request.
